# Hypnosis in Burn Care: Efficacy, Applications, and Implications for Austere Settings

**DOI:** 10.3390/ebj5030020

**Published:** 2024-07-01

**Authors:** Deanna C. Denman

**Affiliations:** US Army Institute of Surgical Research & Burn Center, 3698 Chambers Pass, Ft Sam Houston, TX 78234, USA; deanna.c.denman.civ@health.mil

**Keywords:** hypnosis, non-pharmacological pain management, burns

## Abstract

Burn injuries are among the most traumatic events a person can endure, often causing significant psychological dysfunction and severe pain. Hypnosis shows promise as a complementary intervention to manage pain and reduce the psychological distress associated with burn injury and treatment. This paper reviews the literature regarding hypnosis and potential applications of hypnosis in the management of burns. Hypnosis offers an effective, low-cost intervention that is widely applicable in the management of burns and can play a role in more acute and austere settings where resources are often limited.

## 1. Introduction

Burn injuries can be severe and traumatic and cause significant psychological dysfunction. The pain from the initial injuries and subsequent treatments (e.g., multiple debridement and skin grafting surgeries, dressing changes, and rehabilitation) is a source of distress for patients that contributes to the myriad of associated psychological concerns [1]. Thus, the patient’s psychological treatment needs are complex and span the continuum of care. Clinical hypnosis is well-suited to address some of the needs of burned patients throughout their recovery.

Among burned and traumatically injured patients, the pain experience is multifaceted. Treatment of burn pain must consider both the circumstances of the pain (i.e., its occurrence as background, procedural, breakthrough, or chronic) and the category of pain (i.e., nociceptive, neuropathic, or nociplastic) [2]. Though opioids are the most common treatment of choice, they are limited in their ability to address neuropathic pain and itching and have adverse side effects [3,4]. Multimodal pain management efforts are essential [5].

Evidence is mounting that mind–body therapies, such as hypnosis, have a role in the treatment of major injuries and illnesses. Hypnosis is “a state of consciousness involving focused attention and reductions in peripheral awareness characterized by an enhanced capacity for response to suggestions” [6]. Practically, patients participate in a hypnotic induction with suggestions to achieve relaxation and focus. The hypnotic state, called a trance, allows for increased concentration and suspense of judgmental thinking, which allows for greater reception to positive suggestions. Hypnosis offers a seemingly safe, effective, and low-cost intervention that is widely applicable in pain management [7]. Evidence suggests hypnosis is more effective than other psychological pain management strategies [8,9]. Though care centers are increasingly accepting of mind–body therapies and adjunctive treatments for pain management, hypnosis can also play a role in more acute and austere settings such as the battlefield.

## 2. Review of Applicable Research

Recent research supports the implementation of hypnosis as a nonpharmacologic intervention for burn patients [2,10,11,12,13]. Hypnosis has shown benefits in managing pain, reducing stress and anxiety, and decreasing post-traumatic symptomology. Studies examining hypnosis as an adjunct treatment for burn pain suggest that early intervention with hypnosis can reduce the development of pain and reduce pain and anxiety in patients undergoing wound care and dressing changes [14]. A recent meta-analysis of hypnosis in burns showed statistically significant reductions in pain and anxiety [8]. One review demonstrated the increased benefit of hypnosis over structured attention for reducing pain and anxiety during debridement [15]. Another systematic review demonstrated the benefit of hypnosis in changing perceived pain quality and anxiety in burn patients [16].

Hypnosis has additionally demonstrated benefits in the management of depression [17,18], though this has not specifically been tested in burn patients. One study found the combination of hypnosis with cognitive-behavioral therapy showed benefits over and above those of cognitive-behavioral therapy in isolation [19]. These effects were maintained at 6- and 12-month follow-up assessments. A randomized controlled trial comparing hypnosis to cognitive-behavioral therapy, a gold-standard treatment for depression, found hypnosis was non-inferior to CBT and 44.6% of patients showed a ≥50% reduction in depression symptoms [20].

Studies on applications of hypnosis to trauma-related symptoms are promising. A meta-analysis examined the outcomes of six studies and found hypnosis had a positive impact on PTSD symptoms—particularly avoidance and intrusion [21]. Hypnosis displayed an additive effect with cognitive-behavioral therapy on the symptoms of acute stress disorder when imaginal exposures were preceded by a hypnotic induction [22]. CBT in conjunction with hypnosis resulted in greater reductions in re-experiencing symptoms than CBT alone [22]. Another study found hypnosis was effective in mitigating the re-experiencing symptoms of patients following burn injury [23], highlighting potential benefits for the long-term psychological aspects of burn injuries. Altogether, the evidence collectively underscores the budding applications of hypnosis in managing psychological distress for burn patients.

While the literature on hypnosis shows promising effects, conclusions have been hindered by key challenges in the research [14]. First, most studies examining hypnosis have small sample sizes. Additionally, studies often fail to include appropriate comparators in trials. Finally, hypnosis is rarely standardized, and in fact, is thought to be more effective when tailored, making it difficult to compare effectiveness across studies [13]. These factors often lead to challenges in drawing firm conclusions about the effectiveness of hypnosis. In spite of these difficulties in the research, the American Burn Association has recognized hypnosis as one of the most effective non-pharmacologic interventions for the management of burn pain and endorses its use clinically [24].

## 3. Applications of Hypnosis in Burn Care

Hypnosis has emerged as a promising adjunctive therapy for pain management in burn care, offering unique advantages in addressing the physical and psychological distress experienced by burn patients. Hypnosis can induce analgesic and anesthetic states, effectively reducing pain perception and increasing pain tolerance in burn patients [25,26]. In addition to pain relief, hypnosis can reduce anxiety, fear, and psychological distress associated with injury and treatment [14,26]. Indeed, there is a demonstrated bidirectional relationship between pain and anxiety in medically ill patients: pain worsens anxiety and anxiety increases perceived pain [27]. Through the power of suggestion and imagery, hypnotherapy can modulate pain pathways, alter sensory processing, and promote relaxation, providing relief from burn-related pain and increasing coping [28,29].

The practical application of hypnosis in burn care requires adjustment to the standard process. The standard process of hypnosis in an outpatient setting would involve education, obtaining informed consent, formal assessment of history, symptoms, needs, and careful debriefing. The hypnotic session would also take place in the provider’s office with minimal interruptions. The environment, however, dictates providers must adapt when hypnosis is applied in acute and austere settings. Often care will be provided at bedside and while the patient is in crisis. These challenges result in hypnotic interventions that are brief, focused, and directive [30]. Hypnotic interventions in acute settings may be accomplished in as little as 10–15 min and mitigate pain and anxiety while not extending the time to complete medical procedures [31,32]. Evidence suggests shorter hypnotic interventions are no less effective than longer ones [33,34].

Conceptualizing the use of hypnosis in acute and austere settings has a great deal in common with the principles of psychological first aid [35]. Practitioners must quickly establish rapport, educate clients on relevant concepts, and use brief interventions. Successful hypnosis relies on building trust between the provider and patient to allow for acceptance of the intervention. Rapport is the foundation for the provision of emotional support and help the patient cope in crisis. The provider must then explain the concepts of hypnosis and briefly outline the goal (e.g., “help you be more comfortable.”) By educating the patient, the provider can obtain consent and is able to enhance the patient’s receptiveness and participation in the hypnotic process. Finally, the provider must move into the intervention. The stress of the situation and the requisite distractions often make it difficult for patients to sustain attention. The practitioner may then move into rapid induction and analgesic suggestions to enhance relaxation, mitigate distress, and relieve pain.

### 3.1. Induction

Induction is the process through which a patient is brought into the hypnotic state. The induction is crucial, allowing the patient to enter the hypnotic state, preparing the patient for subsequent interventions (i.e., suggestions), and enhancing patient response [36]. There are many types of inductions ranging in length, focus, and technique. Most experts agree inductions should be personalized to the patient for the best effects (e.g., adjustments should be made based on the patient’s response) and tailored inductions have shown increased effects [37,38]. While a review of various induction strategies is beyond the scope of this paper, common strategies include relaxation, eye fixation or eye roll, and the use of a safe, special, or favorite place.

In addition to bringing the patient into the hypnotic state, the induction functions to build expectancy and motivation, begin seeding suggestions, and bring relief. For example, a relaxation-oriented induction will help alleviate anxiety and distress. Having the patient return to a safe or favorite place can help them begin to separate themselves from their current experience (i.e., dissociate) and experience greater comfort.

### 3.2. Targeted Suggestions

Hypnotic suggestions, the directives given to the patient by the therapist, are the primary means by which the patient changes their perceptions, sensations, thoughts, and behaviors. Well-crafted suggestions are crucial to the successful management of burn patients and may be direct or indirect. Direct suggestions are overt and easily understood, while indirect suggestions are implied and are often metaphorical. Both types of suggestions are helpful in the treatment of burn patients and, ideally, are woven together to reinforce one another. When treating burns and other traumatic injuries, practitioners will most likely focus their suggestions on analgesia/anesthesia, dissociation, time distortion, and mitigating distress (Table 1).

Hypnotic analgesia and anesthesia involve the reduction in pain and other physical sensations without the loss of consciousness. In a hypnotic state, patients can experience reductions in pain intensity, discomfort, and other unpleasant sensations. Research has explored the use of hypnotic analgesia to allow patients to undergo medical procedures without anesthesia or with significant decreases in the required doses of medications. These studies have shown positive outcomes and decreases in complications [31,39].

Hypnotic analgesia can be elicited in a variety of ways. One of the most popular includes the “glove anesthesia” technique, in which the client is given suggestions to create numbness in their hand. This numbness can then be transferred to the parts of the body where numbness is most needed. Alternatively, clients can be given suggestions to find a “pain dial” that they use to “turn down” their pain.

Dissociation refers to a state of detachment from one’s experiences. In hypnosis, dissociation can be elicited to create detachment from distressing sensations and thoughts. Thus, patients can disengage from the painful stimuli of their injuries or their care procedures. Dissociation from pain can be elicited with simple suggestions such as, “your body can be here while your mind is elsewhere.” This may involve suggestions for patients to allow their minds to go to a “safe place”. Dissociation may also be combined with suggestions for time regression that allows clients to revisit a pleasant memory rather than focusing on their current, painful experiences. When discussing dissociation, it is important to differentiate pathologic and nonpathologic dissociation. Dissociation is characterized on a continuum that ranges from daydreaming and absorption to trauma-related dissociation. Though adaptive in some regards, research has connected persistent derealization, the sense of the world not being real, with the development of PTSD [40,41]. Research has also clarified that hypnosis does not activate the pathological aspects of dissociation [42]. In contrast, hypnotically induced dissociation is utilized for therapeutic benefit and is reversed during the re-alerting and reorientation process.

Time distortion alters a client’s perception of time such that it either slows down or speeds up. In hypnosis, both speeding and slowing of time can be helpful. For example, the practitioner may suggest, “time will pass more swiftly during moments of pain”, and “moments of comfort will last longer and longer”, thus reducing anxiety and distress. Practitioners can modulate pain perception by using suggestions to increase comfort and enhance pain tolerance [43,44]. These tools can address pain’s sensory, cognitive, and emotional components, empowering patients to manage their pain experiences.

Implementation of hypnosis may be streamlined using standardized scripts by trained personnel or the use of audio recordings. Patient-tailored inductions, though seen as superior, may not be feasible. Using standardized scripts or pre-recorded audio has advantages when resources are limited. Research by Lang and colleagues utilized a standardized script to induce relaxation at the onset of surgeries and procedures to good effect and significantly reduced procedural pain and anxiety [31,39]. Scripted hypnosis can be personalized and tailored to the individual’s specific fears, anxieties, and pains [31]. Pre-recorded hypnosis has also shown benefits in planned medical procedures [39,45] and may be beneficial for patients during wound care and dressing changes [12].

### 3.3. Assessing Hypnotizability

Hypnotizability, “a person’s ability to experience suggested alterations in physiology, sensations, emotions, thoughts, and behavior during hypnosis”, is a trait with individual differences [6]. Though not always possible in the case of acute pain, a person’s ability to experience hypnosis is measurable. Generally, hypnotizability follows the standard curve, with most people being at least somewhat hypnotizable and the majority of people falling into the moderate range [46]. The degree of benefit seen from hypnotherapy is associated with hypnotizability (i.e., the more hypnotizable one is, the more perceived benefit one will experience from hypnosis). Several measures exist for the formal evaluation of hypnotizability, and these can be helpful in typical settings [46,47,48]. Validated measures of hypnotizability include the Hypnotic Induction Profile [48] (HIP), the Stanford Hypnotic Clinical Scales (SHCS [47]), and the Elkins Hypnotizability Scale (EHS [46]. Administration time ranges from 5 to 30 min.

Though assessment of hypnotizability is the gold standard in clinical practice, it may not be feasible in acute and austere settings. Research has shown, however, that the vast majority of medical patients can benefit from hypnosis [49,50]. In the case of acute pain (such as in the cause of burns and traumatic injuries), patients are in crisis and highly anxious. Hypnosis is well-suited to mitigate anxiety and combat the associated sense of helplessness. Additionally, in emergent and highly stressful situations, there is evidence that people are primed to experience the hypnotic state [51]. Evidence suggests that those with moderate hypnotic suggestibility can experience as much pain relief from hypnosis as those with high hypnotic suggestibility [52]. Taken together, this suggests hypnosis is likely worth attempting even without a formal assessment of hypnotizability in an acute setting. Though patients may differ in the degree of benefit they experience, they are likely to experience some benefit. These differences are seen across all clinical interventions and should not dissuade the provider from the attempt.

### 3.4. Risks of Hypnosis

Though generally seen as safe, there are important risks to consider when applying hypnosis. The most common adverse effects documented by providers were emotional upset, disorientation and drowsiness, memory of traumatic events, and difficulty in being realerted [53]. Recent studies have found that serious adverse effects are uncommon [53,54,55] and nearly half of all providers report having never experienced an adverse effect with their patients [53]. Given the potential for unintended re-experiencing of trauma and dissociative experiences, providers should be aware of common adverse effects, take care to obtain a thorough history and be prepared to assist their patient in recovering.

## 4. Implications for Austere Environments

The application of hypnosis as an adjunct treatment in the care of combat injuries could be multifaceted. Considering both restricted resources and the overall benefit of non-pharmacological treatments, care of combat casualties could be enhanced by hypnosis. Hypnosis could serve as a tool in the immediate aftermath of combat-related injuries as well as in austere environments when resources are limited. Hypnosis offers a non-pharmacological intervention that can be administered with minimal equipment and training, making it well-suited for use in resource-constrained environments. Hypnosis as an adjunctive treatment can help conserve medical resources and enhance the sustainability of medical care in austere environments by minimizing the use of medications with potential side effects and supply chain dependencies.

Hypnosis can also be employed to mitigate the psychological distress associated with injury. The ability of hypnosis to decrease anxiety, depression, and PTSD symptoms would be particularly beneficial in a battlefield setting, where the psychological impact of injuries compounds with that of physical trauma. Hypnosis additionally offers a unique benefit in that it allows providers to empower patients with a tool (i.e., self-hypnosis) they can employ to exert more control over their pain experience, a critical aspect in preventing feelings of helplessness that impact long-term psychological outcomes. Hypnosis represents a promising avenue to augment pain management strategies, improve psychological resilience, and enhance overall patient care in challenging environments. Hypnosis is additionally attractive in austere environments given the range of providers who may be trained in its use (e.g., physicians, nurses, and behavioral health providers).

As combat operational stress control (COSC) units are increasingly deployed, the potential to have a greater number of available providers with the capacity to be trained in hypnosis has increased. Hypnosis is a viable therapeutic intervention for behavioral health providers in a COSC unit to address stress, anxiety, and trauma. Hypnosis may offer an additional self-management skill that patients may be trained to use. Alternatively, personnel in battlefield settings such as medics and technicians who may not practice independently or receive full training to provide hypnotherapy could be trained to use brief hypnotic scripts or offer pre-recorded audio to mitigate pain and anxiety. More clinical trials are needed in burn and trauma patients to elucidate the effects of brief hypnotic interventions and to further validate its application in austere environments.

For those interested in becoming trained hypnosis providers, there are a variety of opportunities for comprehensive training. Training encompasses theoretical knowledge, practical skills, and ethical considerations. Certification is available through multiple reputable organizations, such as the American Society of Clinical Hypnosis (ASCH), the Society of Clinical and Experimental Hypnosis (SCEH), and the International Society of Hypnosis (ISH). All these organizations emphasize the importance of comprehensive education and training as well as ethical practice. Certification through any of the above-listed organizations requires candidates to complete a specified number of training hours, have supervised clinical experience, and demonstrate proficiency in evidence-based techniques, thus leaving providers well-equipped to implement hypnosis into their practice and apply it across a variety of settings.

## 5. Conclusions

The application of hypnosis for burn patients holds significant implications for improving outcomes and enhancing patient comfort. By providing targeted pain relief, reducing anxiety, and promoting psychological well-being, hypnosis complements conventional approaches to burn care. As research continues to elucidate the mechanisms and efficacy of hypnosis in burn care, its integration into clinical practice and austere settings has the potential to augment treatment, offering safe, effective, and compassionate care.

## Figures and Tables

**Table 1:** Types of suggestions.

	Definition	Examples
Analgesia/Anesthesia	Reductions in pain and other physical sensations	- Warmth, or coolness, or numbness… whatever you find most soothing… settling into the place where you most need it
Dissociation	Detachment from one’s experiences (e.g., pain, anxiety, loud noises and distractions)	- Your mind can drift to a time when you felt comfort… this memory, this place slowly coming back to you…
Time Distortion	Alterations in one’s perceptions of time (i.e., time moving more quickly or more slowly)	- Time will pass more swiftly during moments of pain - Moments of comfort will last longer and longer
